# Multiscale simulation approach to investigate the binder distribution in catalyst layers of high-temperature polymer electrolyte membrane fuel cells

**DOI:** 10.1038/s41598-021-04711-9

**Published:** 2022-03-09

**Authors:** Sung Hyun Kwon, So Young Lee, Hyoung-Juhn Kim, Sung-Dae Yim, Young-Jun Sohn, Seung Geol Lee

**Affiliations:** 1 School of Chemical Engineering, Pusan National University, 2, Busandaehak-ro 63beon-gil, Geumjeong-gu, Busan, 46241 Republic of Korea; 2 Hydrogen & Fuel Cell Research Center, Korea Institute of Science and Technology, Hwarang-ro 14-gil 5, Seongbuk-gu, Seoul, 02792 Republic of Korea; 3 Fuel Cell Laboratory, Korea Institute of Energy Research (KIER), Yuseong-gu, Daejeon, 34129 Republic of Korea; 4 Hydrogen Energy Engineering, University of Science and Technology, Yuseong-gu, Daejeon, 34113 Republic of Korea; 5 Department of Organic Material Science and Engineering, Pusan National University, 2, Busandaehak-ro 63beon-gil, Geumjeong-gu, Busan, 46241 Republic of Korea

**Keywords:** Atomistic models, Fuel cells

## Abstract

A multiscale approach involving both density functional theory (DFT) and molecular dynamics (MD) simulations was used to deduce an appropriate binder for Pt/C in the catalyst layers of high-temperature polymer electrolyte membrane fuel cells. The DFT calculations showed that the sulfonic acid (SO_3_^−^) group has higher adsorption energy than the other functional groups of the binders, as indicated by its normalized adsorption area on Pt (− 0.1078 eV/Å^2^) and carbon (− 0.0608 eV/Å^2^) surfaces. Consequently, MD simulations were performed with Nafion binders as well as polytetrafluoroethylene (PTFE) binders at binder contents ranging from 14.2 to 25.0 wt% on a Pt/C model with H_3_PO_4_ at room temperature (298.15 K) and operating temperature (433.15 K). The pair correlation function analysis showed that the intensity of phosphorus atoms in phosphoric acid around Pt (${\rho }_{\mathrm{P}}{g}_{\mathrm{Pt}-\mathrm{P}}\left(r\right)$) increased with increasing temperature because of the greater mobility and miscibility of H_3_PO_4_ at 433.15 K than at 298.15 K. The coordination numbers (CNs) of Pt–P(H_3_PO_4_) gradually decreased with increasing ratio of the Nafion binders until the Nafion binder ratio reached 50%, indicating that the adsorption of H_3_PO_4_ onto the Pt surface decreased because of the high adsorption energy of SO_3_^−^ groups with Pt. However, the CNs of Pt–P(H_3_PO_4_) gradually increased when the Nafion binder ratio was greater than 50% because excess Nafion binder agglomerated with itself via its SO_3_^−^ groups. Surface coverage analysis showed that the carbon surface coverage by H_3_PO_4_ decreased as the overall binder content was increased to 20.0 wt% at both 298.15 and 433.15 K. The Pt surface coverage by H_3_PO_4_ at 433.15 K reached its lowest value when the PTFE and Nafion binders were present in equal ratios and at an overall binder content of 25.0 wt%. At the Pt (lower part) surface covered by H_3_PO_4_ at 433.15 K, an overall binder content of at least 20.0 wt% and equal proportions of PTFE and Nafion binder are needed to minimize H_3_PO_4_ contact with the Pt.

## Introduction

Environmentally friendly energy technology needs to be continuously studied to not only solve environmental problems such as climate change but also satisfy increasing energy demand^1^. Among various energy technologies, fuel cells represent an environmentally friendly energy source that can help alleviate environmental problems. Moreover, fuel cells are promising systems for the noise-free supply of steady energy, with fast start-up and with greater power density compared with fossil fuels^2^. In particular, polymer electrolyte membrane fuel cells (PEMFCs) use a polymeric membrane for proton transport in conjunction with the generation of electricity via the conversion of chemical energy stored in fuels such as hydrogen instead of fossil fuels^3–6^.

PEMFCs are categorized as low-temperature (LT) or high-temperature (HT) PEMFCs depending on their operating temperature^7,8^. LT-PEMFCs have the advantages of higher power densities, quicker start-up, and greater energy conversion efficiencies while generating little noise and operating at temperatures less than 100 °C. However, LT-PEMFCs have disadvantages of poor CO tolerance of the Pt catalyst in their catalyst layers and humidity-dependent performance^9–14^. By contrast, HT-PEMFCs have several advantages related to their higher operating temperature of 100–200 °C. For example, CO poisoning of the Pt catalysts in the catalyst layers of LT-PEMFCs results in substantial degradation of cell performance^15^, whereas HT-PEMFCs are more CO-tolerant than LT-PEMFCs because CO molecules do not easily adsorb onto the Pt surface at elevated temperatures^16^. Moreover, humidification in LT-PEMFCs systems is critical to prevent drying of the polymer membrane; by contrast, HT-PEMFCs exhibit less dependence on humidity than LT-PEMFCs because proton transfer in HT-PEMFCs occurs without water dragging^7,16^. Therefore, HT-PEMFCs can be constructed with a simpler system architecture than LT-PEMFCs^7^. However, HT-PEMFCs have disadvantages of lower energy efficiency than LT-PEMFCs because of the slow kinetics of the oxygen reduction reaction (ORR) and the hydrogen oxidation reaction, which can affect cell performance and lower durability by Pt poisoning and increment of Pt particle size^8,17–21^. Therefore, the commercialization of HT-PEMFCs requires improvements in the cell performance and durability of HT-PEMFCs and a reduction of their manufacturing cost.

HT-PEMFCs comprise gas-diffusion layers, catalyst layers, and polymer-based membranes. The polymer-based membranes are usually polybenzimidazole (PBI)-based membranes doped with phosphoric acid (H_3_PO_4_) to enable proton transport at temperatures greater than the boiling point of water. The catalyst layers consist of a carbon support with a catalyst (typically Pt), a polymer binder, and H_3_PO_4_. The H_3_PO_4_ in the catalyst layers not only performs the same role as the H_3_PO_4_ in the proton-transfer membranes but also participates in electrochemical reactions by adsorbing onto the Pt catalyst. However, carbon corrosion, particle agglomeration, and acid leaching in the catalyst layers of HT-PEMFCs are substantial problems that affect cell performance and durability^20^. The strong adsorption of H_3_PO_4_ onto the Pt surface can adversely affect cell performance by decreasing the oxygen permeability and interfering with the ORR in the catalyst layers^22^. Moreover, the durability of the Pt and the carbon surface in catalyst layers is strongly affected by H_3_PO_4_. Notably, Pt particles have been found to be poisoned by phosphate anions in catalyst layers^23^, and H_3_PO_4_ adsorbed onto the Pt surface adversely affects the ORR^24^. Moreover, the durability of the carbon surface is also affected by the distribution of H_3_PO_4_ in the catalyst layers. The corrosion of the surface of carbon in the cathode was found to be accelerated under prolonged conditions of high temperature, low pH, and high O_2_ concentration^25,26^. Therefore, polymer binders have been used to improve the catalyst layers’ mechanical properties when integrated into the carbon support and catalyst. Manipulating the polymer binder content of the catalyst layers enables control of the hydrophobicity and hydrophilicity^27^. In particular, increasing the polymer binder content can increase the durability by preventing H_3_PO_4_ flooding in the catalyst layers^28^.

Polytetrafluoroethylene (PTFE)^29–37^, polyvinylidene difluoride (PVDF)^29,37^, PBI^29,38–41^, Nafion^29^ and PBI–PVDF blends^29,42,43^ have been mainly used as polymer binders to protect catalyst layers, and the content and type of polymer binder can affect cell performance and durability. Jeong et al.^35^ reported the optimum PTFE binder with 20 wt% of PTFE binder content for maximizing cell performance of HT-PEMFCs. In addition, Su et al.^29^ also reported that ~ 30 wt% of PTFE binder used in HT-PEMFCs. Further investigations of the contents and types of polymer binders are needed to improve cell performance and durability.

Therefore, in the present study, we conducted a molecular dynamics (MD) simulation for the system of catalyst layers in a HT-PEMFC to improve their binding durability. MD simulation methods have been efficiently used to investigate the structures and diffusion properties of H_3_PO_4_ in HT-PEMFC membranes^44,45^. For example, H_3_PO_4_-doped meta-PBI and para-PBI membranes in a HT-PEMFC were investigated, which led to the discovery of a hydrogen bond network for proton transport^44^. In another study, the diffusion coefficients of H_3_PO_4_ and dihydrogen phosphate anion (H_2_PO_4_^−^) in poly(2,5-benzimidazole) (ab-PBI) were investigated with various doping levels of H_3_PO_4_ to elucidate the proton transfer mechanism^45^. Notably, MD simulation methods have been used to investigate the detailed distribution of polymer binders and H_3_PO_4_ in catalyst layers^46,47^. The results of such MD simulations have indicated that a suitable amount of PTFE binder is required to prevent H_3_PO_4_ from reaching the Pt–C surface and thereby improve the durability of the catalyst layers^47^. The adsorption of H_3_PO_4_ onto the Pt surface was found to be substantially increased by an increase in temperature from room temperature (298.15 K) to the operating temperature of HT-PEMFCs (433.15 K). A follow-up study on the selection of polymer binders is needed to minimize carbon corrosion and Pt poisoning at the PEMFC operating temperature.

In the present MD simulation study, we used density functional theory (DFT) calculations to find appropriate binder candidate groups for use in the catalyst layers in HT-PEMFCs. In particular, we analyzed the adsorption energy between the binder candidate groups and the Pt/C surface, which strongly influences durability. Moreover, we discovered that the interactions can affect the distribution morphologies of polymer binders on the Pt/C surface with H_3_PO_4_ and that Nafion binders can protect the carbon surface and Pt particles by preventing excess adsorption of H_3_PO_4_. For this purpose, we used both PTFE and Nafion binders on a Pt/C surface to demonstrate the advantages of each binder in the presence of H_3_PO_4_ at room temperature (298.15 K) and at the HT-PEMFC operating temperature (433.15 K).

## Simulation methods and model preparation

To select the appropriate binder candidates for the Pt/C in catalyst layers, we performed DFT calculations to determine the adsorption energy between the binder candidate polymers and the components of the catalyst layers, such as the carbon surface and the Pt particles. In addition, we performed full-atomistic MD simulations to describe the binder deposit manufacturing process^35^ of the catalyst-layer components, including the Pt/C, H_3_PO_4_, H_2_O, and hydronium (H_3_O^+^) ions, with PTFE and Nafion binder contents ranging from 14.2 to 25.0 wt%.

### DFT simulation for selection of binder candidates

To calculate the adsorption energy of the Pt and carbon surface with binder candidates such as PTFE, PVDF, Nafion, PBI, and ab-PBI, we calculated the DFT adsorption energy using the Vienna ab initio Simulation Package (VASP)^48,49^. The generalized gradient approximation Perdew–Burke–Ernzerhof (GGA-PBE) exchange–correlation functional^50^ was used with projector-augmented-wave (PAW) pseudopotentials^51^ for all geometry optimizations. An energy cut-off of 400 eV was applied with convergence criteria for force (2.0 × 10^−2^ eV/Å) and energy (1.0 × 10^−5^ eV). Moreover, the dipole interaction^52^ along the *z*-axis direction and the DFT-D3 correction of the Grimme scheme^53^ were applied to calculate the adsorption energy between the Pt and carbon surfaces and the binder candidates. The periodic boundary conditions (PBCs) were applied to all directions, and the slab of the Pt and carbon surface was constructed using three atomic layers of a Pt (111) slab and graphite layers with cell sizes of 9.612 × 11.099 × 30.000 Å^3^ and 8.522 × 9.840 × 30.000 Å^3^, respectively. The *k*-points of the Pt (111) slab and the carbon surface were set to a 5 × 5 × 1 and 6 × 5 × 1 Monkhorst–Pack *k*-point meshes^54^ which correspond to the actual spacing of ~ 0.02 Å^−1^ to the x- and y-axis of PBCs, respectively. The adsorption energies (${E}_{\mathrm{adsorption}}$) between the Pt, carbon surface, and binder candidates were calculated using Eq. (1):

1 $${E}_{\mathrm{adsorption}}={E}_{\mathrm{total}}-{E}_{\mathrm{slab}}-{E}_{\mathrm{binder}}$$

where ${E}_{\mathrm{total}}$ represents the total energy of the Pt or carbon surface with the adsorbed binder, ${E}_{\mathrm{slab}}$ represents the total slab energy of the three layers of Pt (111) slab and the graphite layers, and ${E}_{\mathrm{binder}}$ represents the energy of the binder candidates in the PBCs. The adsorption energy of each component of the binder candidates was calculated to predict detailed adsorption mechanisms during binder distribution on the Pt/C surface.

### Force fields and MD simulations

The modified DREIDING force field^55^ was applied to describe the PTFE, Nafion, H_3_PO_4_, and the carbon surface. The H_2_O and the H3O^+^ ions were modeled using an F3C force field^56^ to construct the binder solvent, and a Pt particle was modeled using an embedded atom method (EAM) force field^57^ to describe the catalyst system in a HT-PEMFC. The DREIDING and F3C force fields have been successfully used to describe fuel cell systems^47,58–64^. For the nonbonded interaction between a Pt particle and the Nafion or PTFE binder, we used the nonbonded interaction parameters reported by Brunello et al.^65^; for the nonbonded interaction parameters between a Pt particle and H_3_PO_4_, we used those reported by Kwon et al.^47^ The total potential energies (${E}_{\mathrm{total}}$) of the HT-PEMFC systems were calculated using Eq. (2):

2 $${E}_{\mathrm{total}}={E}_{\mathrm{vdW}}+{E}_{\mathrm{Q}}+{E}_{\mathrm{bond}}+{E}_{\mathrm{angle}}+{E}_{\mathrm{torsion}}+{E}_{\mathrm{inversion}}+{E}_{\mathrm{EAM}}$$

where ${E}_{\mathrm{vdW}}$, ${E}_{\mathrm{Q}}$, ${E}_{\mathrm{bond}}$, ${E}_{\mathrm{angle}}$, ${E}_{\mathrm{torsion}}$, ${E}_{\mathrm{inversion}}$, and ${E}_{\mathrm{EAM}}$ represent the van der Waals, electrostatic, bond-stretching, angle-bending, torsion, inversion, and EAM energies, respectively. The large-scale atomic/molecular massively parallel simulator (LAMMPS) code^66^ from S. Plimpton at Sandia National Laboratory was used to carry out the entire MD simulation for HT-PEMFC systems. The electrostatic interactions were calculated using the particle–particle particle–mesh method^67^. The charges of the atoms in PTFE, Nafion, and H_3_PO_4_ were calculated via Mulliken population analysis^68^ by DFT calculation. All DFT calculations for charge analyses were conducted using a double numerical basis set with polarization (DNP) function and the GGA-PBE^50^ functional using DMol^3^ in the Materials Studio software^69^. The velocity Verlet algorithm^70^ was used with time steps of 0.5 fs and 1.0 fs for evaporation and equilibration MD simulations for integrating equations of atomic motions, respectively.

### Model preparation

To construct the catalyst layers in the HT-PEMFCs, the carbon surface and Pt particles were constructed at full-atomistic scale. The carbon surface was constructed using six graphite layers with 10,752 carbon atoms. In addition, a Pt particle with a diameter of 2.6 nm was constructed using 586 Pt atoms with a truncated octahedral shape with eight (111) planes and six (100) planes because the Pt particles of the commercial TEC10E50E Pt/C (Tanaka Kikinzoku Kogyo, TKK) have a diameter of 2.5 ± 0.4 nm^71^. The weight ratio between the Pt particles and the carbon surface was matched to the commercial Pt/C concentration (45.9 wt% Pt in the TKK catalyst)^35^. The Pt particle was placed on the carbon surface with a PBC of 68.18 × 68.88 × 500.00 Å^3^, and the length of the *z*-direction was set to 500.00 Å with a sufficient vacuum region to prevent interactions beyond the PBC. The chains of the PTFE and Nafion binders were prepared to have 100 and 10 degrees of polymerization (DP), respectively, so that their molecular weight per chain was approximately the same. The PTFE and PTFE–Nafion binder chains were determined to have contents ranging from 14.2 to 25.0 wt%. To prepare the PTFE and PTFE–Nafion binder solvent, 6687 water molecules with 10 additional H_3_O^+^ ions per Nafion chain were used for a solvent. In the binder deposit manufacturing process of the PTFE binder with solvent, an isopropyl alcohol (IPA)–water mixture is typically used to prepare catalyst layers with a PTFE binder^29,33,35,36,72^. Nafion binders have also been deposited using a similar manufacturing process with an IPA–water mixture to prepare catalyst layers^29^. Because the IPA molecules in the IPA–water mixture evaporate more easily than water molecules because of the IPA–water vapor–liquid equilibrium^73^, water molecules eventually remain with the PTFE binders after evaporation of the IPA molecules at a 70:30 (w/w) IPA/water composition^46^. Therefore, we assumed that the water molecules can mainly affect the final dispersion morphology of the PTFE and Nafion binders on the Pt/C surface. Thus, the water molecules were used to construct the PTFE and Nafion solvent to reduce the computational cost. The initial models of PTFE and Nafion solvent with different binder contents were generated by the Monte Carlo method using the Amorphous Cell module in the Materials Studio software^69^.

To obtain equilibrium structures, the PTFE and Nafion solvent models were distributed on a Pt/C surface and canonical ensemble (NVT) MD simulations were performed for 10 ns at 298.15 K. Evaporation simulations in which the temperature was gradually increased from 298.15 to 333.15 K over 30 ps were then performed by NVT simulation, along with an NVT simulation at 333.15 K over 7 ns for evaporation of the water molecules to deposit the chains of PTFE and Nafion binder onto Pt/C surface. The temperature was gradually decreased from 333.15 to 298.15 K over 30 ps and 1 ns of NVT simulation at 298.15 K to complete the evaporation simulations. After the evaporation simulations, 1500 molecules of H_3_PO_4_ were deposited onto a binder–Pt/C surface to construct initial models with H_3_PO_4_, binder, and a Pt/C surface, where the binder contents ranged from 14.2 to 25.0 wt%. After deposition of the H_3_PO_4_ molecules, 15 ns of NVT simulations were performed on equilibrated structures at 298.15 and 433.15 K to analyze the detailed changes in the distribution morphologies of H_3_PO_4_, binder, and the Pt/C surface resulting from a change in temperature. After each equilibration, an additional 5 ns of NVT simulations was performed for data collection. For statistical treatment purposes, at least three independent models were simulated for each case. The detailed molecular composition in our simulations of H_3_O^+^, PTFE binder, Nafion binder, H_3_PO_4_, and Pt/C is summarized in Table 1.

**Table 1:** Compositions of the Pt/C and H_3_PO_4_ with PTFE–Nafion binder in the model systems.

Pt/C	Number of Pt atoms (47.0 wt% in Pt/C)	586				
	Number of C atoms (53.0 wt% in Pt/C)	10,752				
Binder content (wt%)		14.2	17.3	20.0	22.6	25.0
Number of Nafion chains (DP = 10) (*M*_w_ = 9969.83)		2–3	1–3	1–4	1–5	1–6
Number of PTFE chains (DP = 100) (*M*_w_ = 10,039.40)		2–1	4–2	5–2	6–2	7–2
Number of H_3_O^+^ molecules		20–30	10–30	10–40	10–50	10–60
Number of H_3_PO_4_ molecules		1500				

## Results and discussion

### Adsorption properties of binder candidates

**Figure 1:**
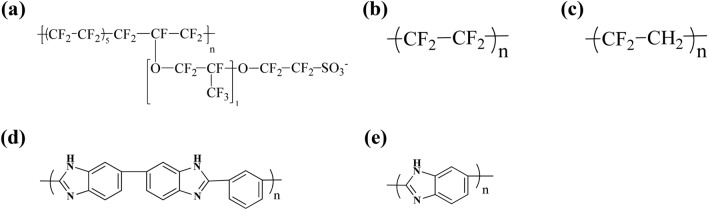
The molecular structures of (**a**) Nafion, (**b**) PTFE, (**c**) PVDF, (**d**) PBI, and (**e**) ab-PBI.

**Figure 2:**
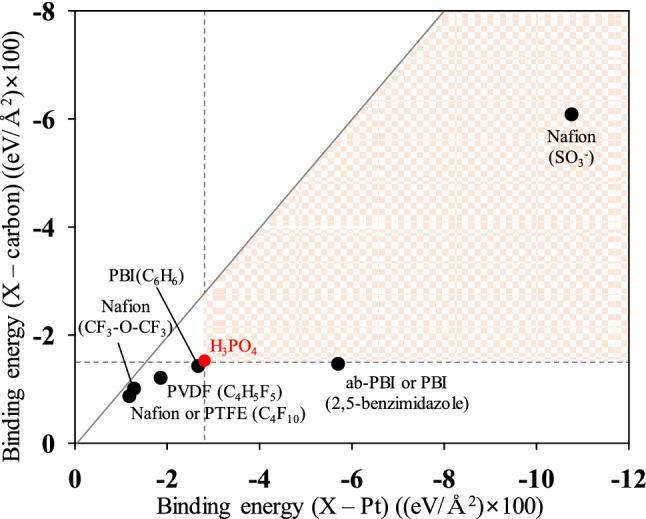
The binding-energy diagram for Nafion, PTFE, PVDF, PBI, and ab-PBI binders.

The binder–Pt, binder–carbon surface, H_3_PO_4_–Pt, and H_3_PO_4_–carbon surface binding energies strongly affect the distribution of H_3_PO_4_ on the Pt/C surface^47^. In particular, to prevent excessive contact of H_3_PO_4_ on the Pt surface and carbon corrosion in the catalyst layers, the binder–Pt and binder–carbon surface adsorption energies should be greater than the H_3_PO_4_–Pt and H_3_PO_4_–carbon surface adsorption energies to prevent excess leaching of the carbon surface and Pt particles. We therefore performed DFT calculations to determine and compare the adsorption energies of various binders. Figure 1 shows the molecular structures of the binders, which are commonly used as binders in the catalyst layers of HT-PEMFCs. We prepared the main component in binders for DFT calculations to compare their binding energies on Pt and the carbon surface. In particular, the Nafion (CF_3_–O–CF_3_, CF_3_–SO_3_^−^, C_4_F_10_), PTFE (C_4_F_10_), PVDF (C_4_H_5_F_5_), PBI (benzene, 2,5-benzimidazole), ab-PBI (2,5-benzimidazole) components were calculated on Pt and graphite layers. The results of the binding energies are shown in Table 2. We also normalized the binding energies according to the adsorption areas to enable a quantitative comparison. Figure 2 shows the binding-energy diagram for selecting appropriate binder candidates by comparing the adsorption energy between Pt and the carbon surface on the basis of the adsorption energy of H_3_PO_4_ (Table 2). The binding energy of H_3_PO_4_ on the Pt and carbon surfaces is − 0.0281 eV/Å^2^ and − 0.0151 eV/Å^2^, respectively. The appropriate binder candidates need to have stronger binding energy than H_3_PO_4_ on the Pt and carbon surfaces to prevent permeation of H_3_PO_4_ into the binders. For example, the binding energies between the PTFE binder and the Pt surface (− 0.0118 eV/Å^2^) and between the PTFE binder and the carbon surface (− 0.0085 eV/Å^2^) are lower than those between H_3_PO_4_ and the Pt surface and between H_3_PO_4_ and the carbon surface. These results mean that the PTFE binder does not readily prevent excessive leaching of the Pt and carbon surfaces in the presence of H_3_PO_4_ at the HT-PEMFC operating temperature. Therefore, the H_3_PO_4_ can contact the Pt surface by permeating into the PTFE binder at the HT-PEMFC operating temperature. However, the binding energies between the sulfonic acid groups (SO_3_^−^) and the Pt surface (− 0.1078 eV/Å^2^) and between the SO_3_^−^ groups and the carbon surface (− 0.0608 eV/Å^2^) are much greater than the corresponding H_3_PO_4_ binding energies. Thus, the SO_3_^−^ groups in Nafion may prevent excess leaching of the Pt and carbon surfaces, thereby improving the mechanical performance and cell durability at the HT-PEMFC operating temperature. We therefore performed MD simulations to investigate the possibility of improving the durability of catalyst layers by controlling the distribution of H_3_PO_4_ by varying the binder content.

**Table 2:** The binding energy of components in binders on Pt(111) and carbon surfaces.

Materials		Pt(111) surface		Carbon surface	
		Binding energy (eV)	Binding energy (eV/Å^2^)	Binding energy (eV)	Binding energy (eV/Å^2^)
Nafion	CF_3_–O–CF_3_	− 0.362	− 0.0128	− 0.285	− 0.0099
	CF_3_–SO_3_^−^	− 2.537	− 0.1078	− 1.409	− 0.0608
Nafion and PTFE	C_4_F_10_	− 0.433	− 0.0118	− 0.313	− 0.0085
PVDF	C_4_H_5_F_5_	− 0.635	− 0.0186	− 0.404	− 0.0119
ab-PBI and PBI	ab-PBI	− 2.456	− 0.0571	− 0.640	− 0.0145
	C_6_H_6_ (benzene)	− 0.937	− 0.0267	− 0.488	− 0.0141
H_3_PO_4_	H_3_PO_4_	− 0.618	− 0.0281	− 0.329	− 0.0151

### Visualization of evaporated Pt/C structures with binders

**Figure 3:**
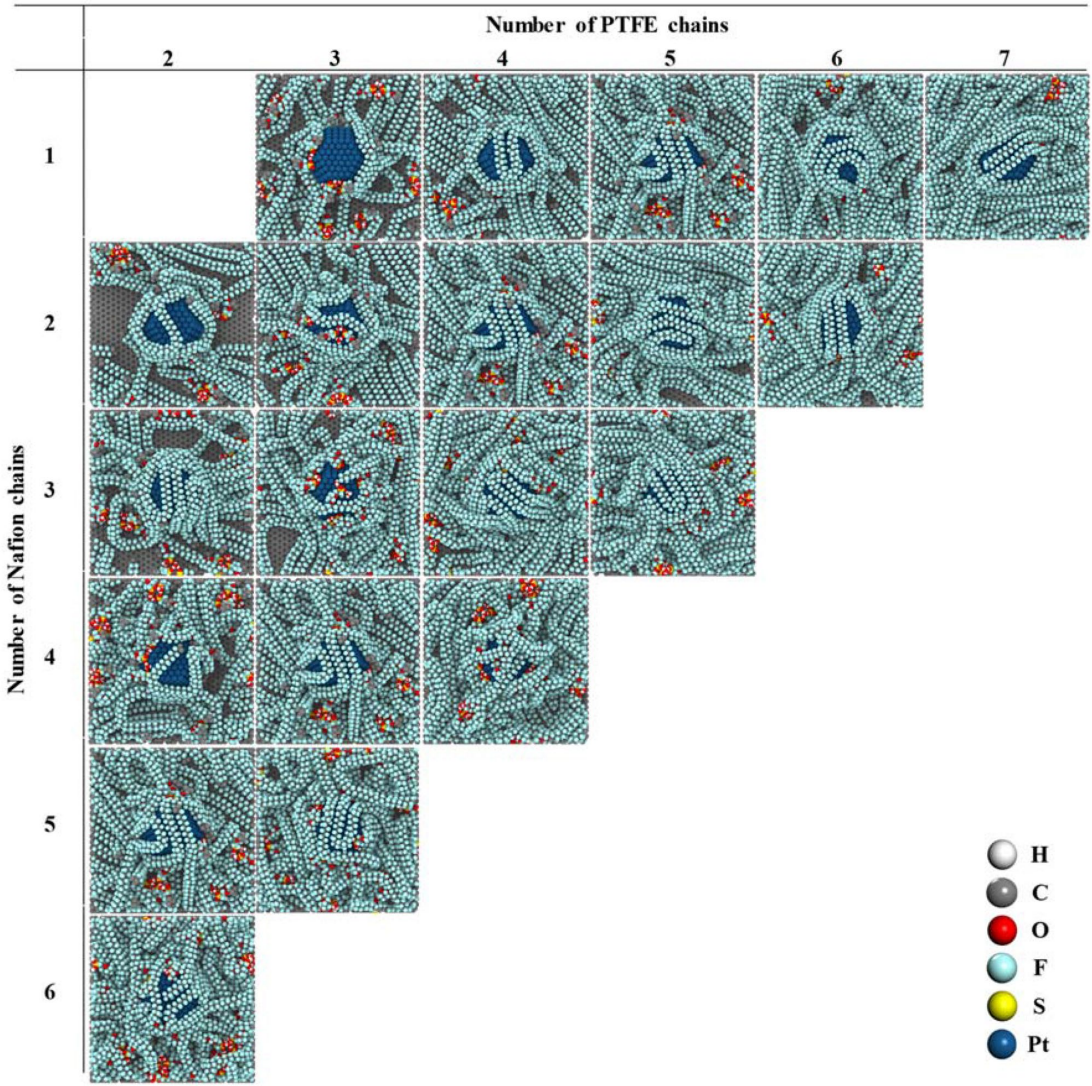
The top view of models after solvent evaporation with various contents (14.2 to 25.0 wt%) of PTFE and Nafion binders on a Pt/C surface. White, gray, red, cyan, yellow, and blue spheres represent hydrogen, carbon, oxygen, fluorine, sulfur, and platinum atoms, respectively.

Figure 3 shows snapshots of the structures after the evaporation MD simulations. The *x*- and *y*-axes indicate the number of PTFE and Nafion chains, respectively, on the Pt/C surface. At the first stage, we conducted the MD simulations with only Nafion binder deposited onto the Pt/C surface to protect both the Pt and the carbon surface by minimizing the adsorption of H_3_PO_4_. However, the Nafion binder tended to predominantly locate on the Pt surface because of the strong adsorption energy between the Pt and the SO_3_^−^ groups of the Nafion chains. Doo et al.^62^ have reported experimental data showing that Nafion strongly adsorbs onto the Pt surface. Thus, Nafion binders can possibly prevent the excess adsorption of H_3_PO_4_ onto the Pt surface. However, preventing the excess adsorption of H_3_PO_4_ onto the carbon surface is difficult because of the strong Nafion–Pt interaction. Notably, the authors of another study^47^ revealed that a sufficient amount of PTFE binder can prevent the adsorption of H_3_PO_4_ onto a carbon surface. It means that not only binding energies between PTFE binders and carbon surface, but also distribution procedure is important to form hydrophobicity surface while PTFE binders were preferentially distributed on carbon surface than H_3_PO_4_. Therefore, not only Nafion but also PTFE binders can be used on Pt/C surfaces to protect both the Pt surface and the carbon surface in the catalyst layers.

As shown in Fig. 3, the influence of the PTFE and Nafion binders was easily identified at low binder contents. For example, simulations for an overall binder content of 14.2 wt% on the Pt/C surface show that more polymer binder with 2 PTFE/2 Nafion chains was attached near the Pt surface than polymer binder with 3 PTFE/1 Nafion chains. By contrast, simulations for an overall binder content of 14.2 wt% also show that more binder with 3 PTFE/1 Nafion chains was attached onto the carbon surface than binder with 2 PTFE/2 Nafion chains. These distribution features show that the PTFE-containing binders positively affect the carbon surface coverage and that the Nafion-containing binders positively affect the Pt surface coverage. With increasing binder content, the Pt and carbon surfaces were gradually covered by PTFE and Nafion binders and each Nafion and PTFE binder was mainly located near the Pt surface and carbon surface, respectively.

**Figure 4:**
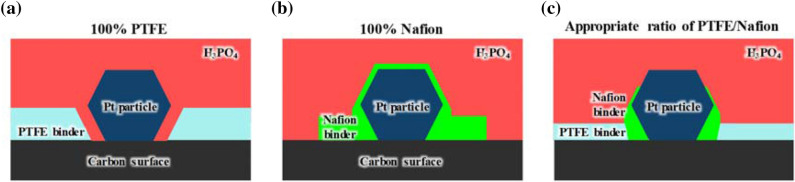
Schematics of binder distribution features for (**a**) 100% PTFE, (**b**) 100% Nafion, and (**c**) an appropriate ratio of PTFE/Nafion binders on the Pt/C surface in a catalyst layer.

We propose a scheme for the distributions of PTFE and Nafion binders on the Pt/C surface. As shown in Fig. 4a, 100% PTFE binder on the Pt/C surface well covers the carbon surface; however, H_3_PO_4_ still contacted between carbon and Pt particle surface by pushing out the PTFE binder which located near carbon surface and Pt particles. Thus, although the PTFE binder well protects the carbon surface against carbon corrosion by H_3_PO_4_, excess PTFE binder is needed to protect the Pt against poisoning by excess adsorption of H_3_PO_4_ onto the Pt surface. On the contrary, as shown in Fig. 4b, 100% Nafion binder well protects the Pt surface, whereas the Nafion binder poorly protects the carbon because of agglomeration of the SO_3_^−^ groups and strong interaction with the Pt. Therefore, the appropriate PTFE/Nafion binder ratio (Fig. 4c) to protect both the Pt and the carbon surface needs to be determined. Moreover, the total contents of the polymer binders should be reduced to prevent a reduction of the electrical conductivity in the catalyst layers because of the electrical insulating character of the PTFE binder and also to determine the optimum binder content^35^. Therefore, the distribution characteristics of H_3_PO_4_ as a function of the contents and ratio of PTFE/Nafion binders on the Pt/C surface were evaluated to deduce appropriate structures for improving the durability of HT-PEMFC catalyst layers.

### Distribution of H_3_PO_4_ on Pt/C structures with binders

For catalyst layers consisting of Pt/C and a polymer binder with H_3_PO_4_, the distribution of H_3_PO_4_ strongly influences their durability. In particular, excess contact between H_3_PO_4_ and Pt can cause undesirable effects such as Pt poisoning and carbon corrosion^20,23,25,26^. Therefore, understanding the distributions of H_3_PO_4_ molecules on the Pt/C surface with PTFE and Nafion–PTFE binders is important for establishing a balance between performance and durability.

**Figure 5:**
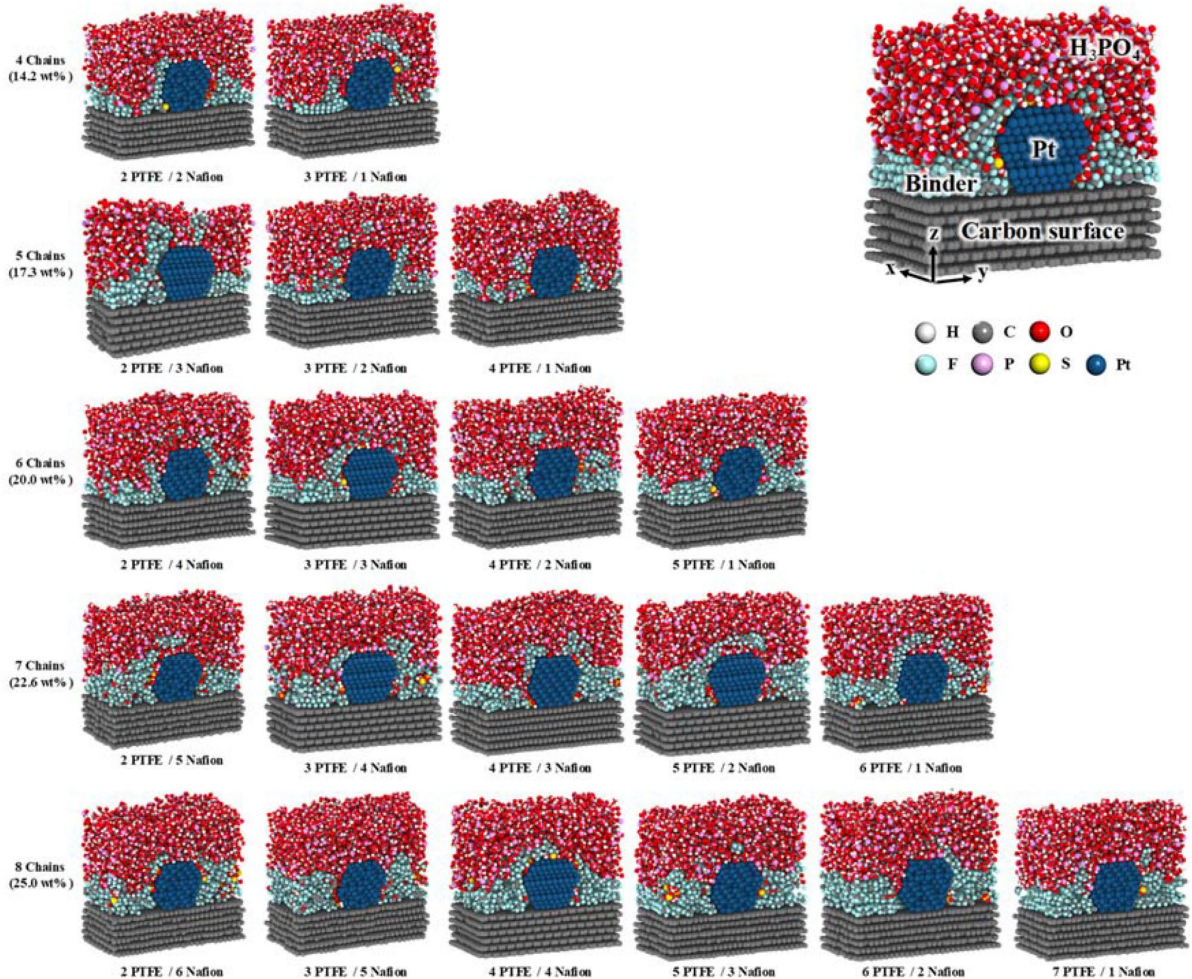
Cross-sectional snapshots of equilibrated Pt/C structures with H_3_PO_4_ and various contents and ratios of PTFE/Nafion binders at 298.15 K. White, gray, red, cyan, pink, yellow, and blue colors represent hydrogen, carbon, oxygen, fluorine, phosphorus, sulfur, and platinum atoms, respectively.

Figure 5 shows cross-sectional snapshots of equilibrated Pt/C structures with H_3_PO_4_ and with PTFE and Nafion binders at contents of 14.2 to 25.0 wt% at 298.15 K. The binders initially covered near the Pt and carbon surface. The binders which are located between Pt and carbon surface can protect the Pt particle and carbon surface by preventing excess adsorption of H_3_PO_4_ onto the Pt and carbon surfaces. Notably, the distributions of the Nafion and PTFE binders were easily distinguished at low binder contents of 14.2 and 17.3 wt%, where the SO_3_^−^ groups in the Nafion were mainly located near the Pt surface, whereas the PTFE was mainly located near the carbon surface. For example, at a binder content of 17.3 wt%, the 2 PTFE/3 Nafion binder showed greater contact with the Pt surface than the 4 PTFE/1 Nafion binder. As the binder content was increased, both the PTFE and the Nafion gradually encapsulated the Pt particle and the carbon surface (Fig. 5).

**Figure 6:**
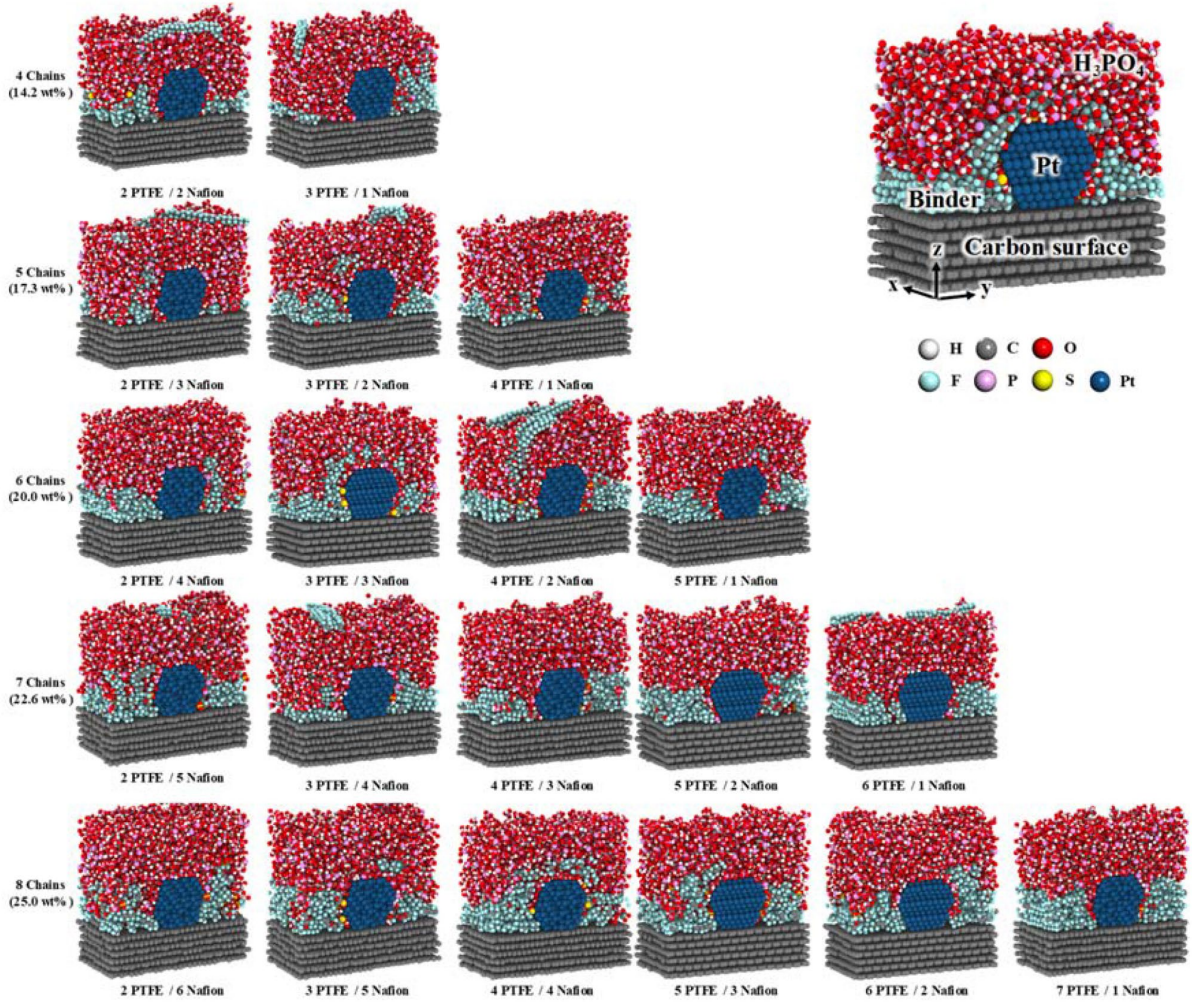
Cross-sectional snapshots of equilibrated Pt/C structures with H_3_PO_4_ and various contents and ratios of PTFE/Nafion binders at 433.15 K. White, gray, red, cyan, pink, yellow, and blue colors represent hydrogen, carbon, oxygen, fluorine, phosphorus, sulfur, and platinum atoms, respectively.

As the temperature was increased from 298.15 K to 433.15 K (HT-PEMFC operating temperature) in Fig. 6, the H_3_PO_4_ molecules permeated into the binders and contacted the Pt surface. In particular, a high proportion of the PTFE binder was permeated by H_3_PO_4_ molecules at 433.15 K because H_3_PO_4_ molecules at this temperatures exhibit greater mobility and miscibility than those at 298.15 K^47^ and because the binders located near the Pt and carbon surface was difficult to maintain at this higher temperature. However, the SO_3_^−^ groups in the Nafion binders were still located near the Pt surface even after the increase in temperature because the SO_3_^−^ groups have a higher adsorption energy than H_3_PO_4_ on the Pt surface.

#### Structural analysis

The pair correlation functions (PCFs) were evaluated to elucidate the different effects of the PTFE and Nafion binders. The PCF equation is

3 $${g}_{A-B}\left(r\right)= \left(\frac{{n}_{B}}{4\uppi {r}^{2}\Delta r}\right)/\left(\frac{{N}_{B}}{V}\right)$$

where ${g}_{A-B}(r)$ indicates the probability of finding atom *A* at distance *r* from atom *B* in the MD simulations for data collection; ${n}_{B}$ is the number of *B* atoms located at distance *r* in a shell of thickness ∆*r* from atom *A*; ${N}_{B}$ and *V* indicate the total number of *B* atoms in the system and the total volume of the system, respectively; and ${N}_{B}/V$ indicates the number density of atom *B*, ${\rho }_{B}$. The ${{\rho }_{B}g}_{A-B}\left(r\right)$ values were used instead of the ${g}_{A-B}\left(r\right)$ values for direct comparisons.

**Figure 7:**
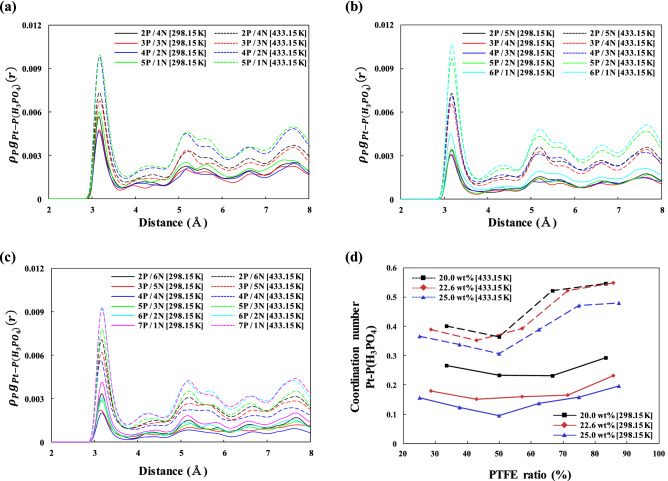
Pair correlation functions (*ρ*_P_*g*_Pt–P_(*r*)) of Pt–P(H_3_PO_4_) at (**a**) 20.0, (**b**) 22.6, and (**c**) 25.0 wt% binder contents with different ratios of PTFE and Nafion binders at 298.15 and 433.15 K. P and N in the legends in (**a**), (**b**), and (**c**) indicate the number of chains of PTFE and Nafion, respectively. (**d**) The coordination numbers (CNs) of Pt–P(H_3_PO_4_) pairs with different ratios of PTFE and Nafion binders at 298.15 and 433.15 K.

Figure 7a–c shows the PCFs of Pt with H_3_PO_4_ at binder contents of 20.0, 22.6, and 25.0 wt%, respectively. All intensities of ${\rho }_{P}{g}_{\mathrm{Pt}-\mathrm{P}}\left(r\right)$ increased with increasing temperature from 298.15 to 433.15 K, indicating that the H_3_PO_4_ molecules were more strongly correlated with the Pt surface at the higher temperature of 433.15 K because the H_3_PO_4_ molecules exhibit greater mobility and greater miscibility with the PTFE binder^47^. To quantitatively analyze the distribution of H_3_PO_4_ molecules on the Pt particles as functions of the temperature and the binder ratio, the first coordination numbers (CNs) of Pt–P(H_3_PO_4_) are shown in Fig. 7d. The CNs were calculated by integrating the first peaks of the intensities of ${{\rho }_{\mathrm{P}}g}_{\mathrm{Pt}-\mathrm{P}}\left(r\right)$ in Fig. 7a–c. The intensities of ${{\rho }_{\mathrm{P}}g}_{\mathrm{Pt}-\mathrm{P}}\left(r\right)$ decreased as the Nafion ratio was increased to 50% (PTFE 50%). This result means that the adsorption of H_3_PO_4_ onto the Pt surface decreased with increasing Nafion binder content because of the high adsorption energy between SO_3_^−^ groups and Pt. However, the intensities of ${{\rho }_{\mathrm{P}}g}_{\mathrm{Pt}-\mathrm{P}}\left(r\right)$ increased as the Nafion ratio was increased beyond 50% (< PTFE 50%) because the excess Nafion binder agglomerated with itself via its SO_3_^−^ groups. Compared with the CNs of Pt–P(H_3_PO_4_) at 433.15 K, those at 298.15 K slowly decreased with decreasing PTFE binder ratio (increasing Nafion binder ratio) until the ratio reached 50%. This result means that the Nafion binder more strongly affected the distribution of H_3_PO_4_ on the Pt surface at 433.15 K than at 298.15 K. Therefore, the distribution of H_3_PO_4_ molecules in the catalyst layers was apparently more sensitive to the Nafion content than to the PTFE content.

#### Surface coverage analysis

**Figure 8:**
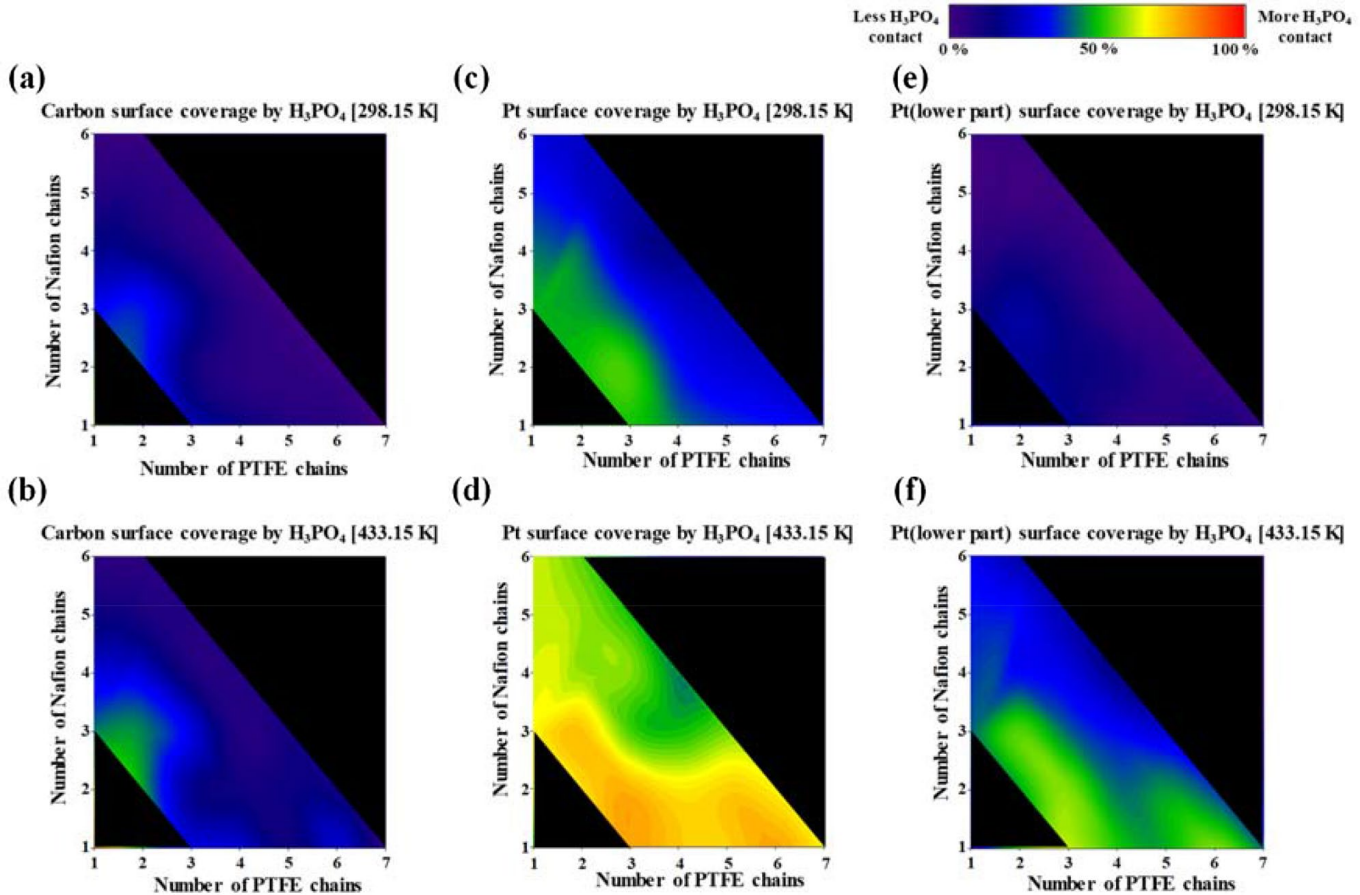
Carbon surface coverage by H_3_PO_4_ at (**a**) 298.15 K and (**b**) 433.15 K. Pt surface coverage by H_3_PO_4_ at (**c**) 298.15 K and (**d**) 433.15 K, and the Pt (lower part) surface coverage by H_3_PO_4_ at (**e**) 298.15 K and (**f**) 433.15 K.

Not only the distribution of H_3_PO_4_ on the Pt surface but also its distribution on the carbon surface strongly affects cell durability in HT-PEMFCs because of Pt poisoning and carbon corrosion. Therefore, we also conducted surface analyses to investigate the detailed distribution of H_3_PO_4_ on the Pt and carbon surfaces as functions of the contents and ratio of PTFE and Nafion binders at 298.15 and 433.15 K. The equation for surface coverage is

4 $${\text{Surface}}\;{\text{Coverage }}\left( \% \right) \, = \, \left( {S_{{{\text{contact}}}} /S_{{{\text{surface}}}} } \right) 100$$

where *S*_contact_ represents the number of Pt or C atoms in contact with H_3_PO_4_ molecules and *S*_surface_ represents the number of Pt or C atoms at the surface of the Pt particle or carbon layer. The contact atoms were counted under the first peak distance of each PCF. Figure 8a,b shows the carbon surface coverage by H_3_PO_4_ at 298.15 and 433.15 K, respectively. The distribution of H_3_PO_4_ molecules on the carbon surface differs depending on the ratio of PTFE and Nafion binders when the binder content is 14.2 wt%. For example, the carbon surface coverage by H_3_PO_4_ at 14.2 wt% with a high ratio of PTFE (3 PTFE chains and 1 Nafion chain) is lower than that at 14.2 wt% with a high ratio of Nafion (1 PTFE and 3 Nafion chains). These results indicate that the H_3_PO_4_ molecules were less distributed on the carbon surface with a high ratio of PTFE binders at the same binder content because the PTFE binder was better distributed than the Nafion binder on the carbon surface. Binders with a high ratio of PTFE have the advantage of protecting the carbon surface by preventing the adsorption of H_3_PO_4_ molecules. By contrast, the Pt surface coverage by H_3_PO_4_ decreased with increasing Nafion ratio at the same binder content because the Nafion binder tended to locate near the Pt surface because of the strong interactions between the Nafion SO_3_^−^ groups and the Pt. The carbon surface coverage by H_3_PO_4_ decreased with increasing overall binder content, and no substantial difference was observed in the H_3_PO_4_ coverage at 298.15 and 433.15 K. Therefore, a binder with at least 20.0 wt% PTFE content should be applied to the Pt/C surface to protect the carbon surface against H_3_PO_4_ under both 298.15 and 433.15 K temperature conditions. These results were well agreed with experimental data which showed the optimized contents of 20.0–30.0 wt% of PTFE binder^29,35^ or ~ 23.0 wt% of Nafion binder^29^.

Figure 8c,d shows the Pt surface coverage by H_3_PO_4_ at 298.15 and 433.15 K, respectively. The Pt surface coverage by H_3_PO_4_ at 298.15 K decreased with increasing binder content. Notably, at 25.0 wt% binder content, the Pt surface coverage by H_3_PO_4_ reached the lowest value when the PTFE/Nafion binder ratio was equal (4 PTFE/4 Nafion). This result means that the same ratio of PTFE and Nafion binders with at least 25.0 wt% binder content provided sufficient coverage of both the Pt and the carbon surface to prevent excess adsorption of H_3_PO_4_. At 433.15 K, binders with a higher ratio of PTFE showed a higher Pt surface coverage by H_3_PO_4_ than binders with the same ratio of PTFE and Nafion because the PTFE binders were easily permeated by H_3_PO_4_ as a consequence of the greater mobility of H_3_PO_4_ and because the adsorption energy of H_3_PO_4_ on Pt is greater than that of PTFE on Pt. Vice versa, at 433.15 K, binders with a high ratio of Nafion led to lower Pt surface coverage by H_3_PO_4_ than binders with the same ratio of PTFE and Nafion because the Nafion binder agglomerated with itself via its SO_3_^−^ groups.

We further analyzed the lower Pt surface coverage by H_3_PO_4_ molecules in Fig. 8e,f because the binders tended to be initially distributed between the lower part of Pt (under half of the Pt layers in Pt particle) and the carbon surface^46^. H_2_O molecules near the Pt surface were generated by the reaction of O_2_ molecules with protons, and the carbon surfaces in the cathode were corroded in the presence of H_2_O under low-pH conditions^26,74^ because the H_2_O molecules near the lower part of the Pt surface promoted contact between the carbon surface and the H_3_PO_4_. At 298.15 K, the lower part of the Pt surface was covered with fewer H_3_PO_4_ molecules than the upper part of the Pt surface because the binders were mainly distributed between the carbon surface and the Pt^46,47^. At 433.15 K, the binders with a higher ratio of PTFE did not prevent the Pt surface from contacting H_3_PO_4_ compared with the binders with other ratios because of the lower adsorption energy of PTFE on the Pt surface compared with that of H_3_PO_4_ on the Pt surface. As the Nafion content in the binders increased at the same overall binder content, the Pt (lower part) surface coverage by H_3_PO_4_ decreased because of less adsorption of H_3_PO_4_ onto the lower part of the Pt surface. Therefore, an overall binder content of at least 20.0 wt% with equal PTFE and Nafion contents should be used to protect the lower part of the Pt surface against H_3_PO_4_ at 433.15 K and thereby improve the durability of the catalyst layers in HT-PEMFCs by protecting the carbon and Pt surfaces.

## Conclusions

DFT calculations were performed to determine appropriate binder candidates for protecting Pt/C catalyst layers and thereby improving cell durability. The SO_3_^−^ groups in Nafion appear to make Nafion an appropriate binder candidate because the SO_3_^−^–Pt and SO_3_^−^–carbon surface binding energies are greater than the H_3_PO_4_–Pt and H_3_PO_4_–carbon surface binging energies. Consequently, we performed full-atomistic MD simulations for binders with various contents (14.2 to 25.0 wt%) of Nafion and PTFE combined in different ratios. The Nafion binders were mainly located near the Pt surface because of higher binding energy of SO_3_^−^ groups with the Pt surface. The intensities of ${\rho }_{\mathrm{P}}{g}_{\mathrm{Pt}-\mathrm{P}}\left(r\right)$ increased with increasing temperature from 298.15 to 433.15 K. At 298.15 K, the coordination numbers of Pt–P(H_3_PO_4_) decreased more slowly than at 433.15 K with decreasing PTFE binder ratio (increasing Nafion binder ratio) until the ratio reached 50%. The carbon surface coverage by H_3_PO_4_ almost converged at binder contents greater than 20 wt% at both 298.15 and 433.15 K. The Pt surface coverage by H_3_PO_4_ was lowest in the case of binders with the same ratio of PTFE and Nafion and at a total binder content 25.0 wt%. For the Pt (lower part) surface coverage by H_3_PO_4_ at 433.15 K, binders with the same contents of PTFE and Nafion and an overall binder content of at least 20.0 wt% were needed to minimize the H_3_PO_4_ contact. We expect that our multiscale approach will aid in the selection of other binder candidates for improving the cell durability and the performance of the catalyst layers in HT-PEMFCs.
